# Isolation of *Elizabethkingia* spp. from Diagnostic Specimens from Dogs and Cats, United States, 2019–2021

**DOI:** 10.3201/eid2907.230218

**Published:** 2023-07

**Authors:** J. Scott Weese, Kurtis E. Sobkowich, Zvonimir Poljak, Theresa M. Bernardo

**Affiliations:** University of Guelph, Guelph, Ontario, Canada

**Keywords:** Elizabethkingia, bacteria, zoonoses, veterinary medicine, infectious diseases, dogs, cats, United States, Canada

## Abstract

We retrospectively reviewed *Elizabethkingia* spp. culture and susceptibility results from 86 veterinary diagnostic laboratory results from US dogs and cats. We noted 26 *E. menigoseptica*, 1 *E. miricola*, and 59 unspeciated *Elizabethkingia* isolates from 9 US states (2–22 isolates per state). *Elizabethkingia* infections in animals might increase risks to humans.

*Elizabethkingia* is a genus of environmental gram-negative bacteria that can cause severe opportunistic infections in humans. The 3 main *Elizabethkingia* species are *E. meningoseptica*, the most common cause of disease; *E. miricola*; and *E. anophelis* (*1*). Human infections are rare—5–10 infections are reported annually per state in the United States (*2*)—but mortality rates can be high, 24%–41% (*1*,*3*,*4*).

*Elizabethkingia* infections have rarely been reported in domestic animals; 1 case was reported in a dog in Portugal (*5*), and 2 isolates were reported from horses in the United States (*6*). We describe *Elizabethkingia* spp. isolated from specimens from dogs and cats submitted to a US diagnostic veterinary laboratory for bacterial culture and susceptibility testing.

We evaluated bacterial culture results from specimens from dogs and cats that were submitted to IDEXX Laboratories (https://www.idexx.com) in the United States during 2019–2021. Available metadata included year collected, state, county, animal species, animal age, anatomic sample site, and antimicrobial susceptibility. Isolates were identified by using MALDI Biotype matrix-assisted laser desorption/ionization time-of-flight mass spectrometry (Bruker Corporation, https://www.bruker.com). Antimicrobial susceptibility was determined by using Clinical and Laboratory Standards Institute (CLSI) breakpoints for non-Enterobacterales bacteria (*7*).

In all, we investigated 86 *Elizabethkingia* spp. isolates: 26 (30%) were *E. meningoseptica*, 1 was *E. miricola*, and 59 (69%) were only identified to the genus level. All isolates were from individual animals; 71 (83%) were from dogs and 15 (17%) were from cats. Twenty-one (24%) isolates were identified in 2019, 36 (42%) in 2020, and 29 (34%) in 2021. Isolates were from 9 states, each of which had 2 (South Carolina, Tennessee) to 22 (Washington) isolates (Table 1). The most common specimen sites were skin and soft tissue infection (25; 29%), abscesses (20; 23%), ears (12; 14%), lower respiratory tract (10; 12%), nasal (6; 7.0%), and surgical site infections (3; 3.5%). We also assessed antimicrobial susceptibility data (Table 2).

**Table 1:** *Elizabethkingia* spp. isolated from diagnostic specimens from dogs and cats, United States, 2019–2021

State	No. isolates
Washington	22
Virginia	13
Pennsylvania	13
Oregon	11
Texas	11
Wisconsin	9
California	3
South Carolina	2
Tennessee	2

**Table 2:** Antimicrobial susceptibility of *Elizabethkingia* spp. isolated from 71 dog and 15 cat diagnostic specimens, United States, 2019–2021*

Antimicrobial drug	No. tested	No. (%) susceptible
Amikacin	84	13 (15)
Amoxicillin	57	4 (7.0)
Amoxicillin-clavulanic acid	57	5 (8.8)
Cefotaxime	46	6 (13)
Cefovecin	57	5 (8.8)
Cefpodoxime	57	7 (12)
Ceftazidime	86	6 (7.0)
Ceftiofur	53	9 (17)
Cephalexin	57	3 (5.3)
Chloramphenicol	64	9 (14)
Doxycycline	59	39 (66)
Enrofloxacin	86	73 (85)
Gentamicin	85	17 (20)
Imipenem	86	7 (8.1)
Marbofloxacin	86	75 (87)
Trimethoprim/sulfamethoxazole	54	48 (89)

*Antimicrobial susceptibility testing performed according to Clinical and Laboratory Standards Institute guidelines (*7*) at IDEXX Laboratories (https://www.idexx.com).

We assessed clustering at the county level over time. We noted 19 counties that had multiple isolates; 4 pairs of isolates at the county level were from specimens submitted within the same month, and another pair of isolates was submitted from a single county in subsequent months. 

Although reports of *Elizabethkingia* spp. infections in animals have been limited, our data indicate that this bacterium is rare but extant in clinical specimens from dogs and cats in the United States. Noninvasive infections predominated; skin infections, abscesses, and wound infections accounted for >50% of isolates. The distribution of infection sites is consistent with an environmental opportunist, for which infection would develop after environmental contamination of compromised sites, particularly after skin barrier damage. Those animal infections contrast with human infections, in which meningitis and bacteremia are most common (*1*,*3*). Whether those differences are because of a true difference in disease distribution or because human data are biased due to more testing of high-risk populations, such as infants and immunocompromised persons, publication biases toward reporting severe disease, or both, remains unclear.

Isolates were from multiple states. Geographic distribution of infection in humans is not well reported in the United States; however, Wisconsin was the site of a notable high incidence outbreak in humans during 2015–2016 (*8*). Further study of geographic patterns in humans and domestic animals is warranted.

Most isolates appeared to be from sporadic infections. In a few instances, 2 isolates were from the same county in the same or subsequent months. Because clinic-level data were not available, whether those isolates were from the same clinics or had any epidemiologic links is unclear. Therefore, although clustering in clinics is possible, as seen in human healthcare facilities, we could not determine whether any of these infections were linked. Because clinical data were not available, we could not determine whether *Elizabethkingia* was the cause of disease or was a clinical contaminant.

The zoonotic risks posed by animals with *Elizabethkingia* spp. infections are unknown; however, 2 equine-origin *E. anopheles* isolates clustered within a clade of human isolates in 1 instance (*6*), and another instance had a similar overlap between isolates from a frog and a human (*9*). Those findings are not unexpected for infections that likely originate in the environment but do not clarify whether zoonotic transmission can occur once an animal has a clinical infection.

*Elizabethkingia* isolates tend to have intrinsic resistance to multiple antimicrobial drugs (*10*). The high prevalence of susceptibility to potentiated sulfonamides (89%) and fluoroquinolones (85%–87%) for samples from dogs and cats in this study is consistent with human data (*10*), as would be expected if a common environmental source was involved.

Although rare, *Elizabethkingia* spp. were identified in dogs and cats in multiple US states. Because *Elizabethkingia* is an environmental pathogen, human and animal exposures presumably are from similar environmental sources. Thus, an understanding of infections in animals might have relevance for assessing risks to humans and for identifying potential animal health risks.
